# Prescribed-Time Leader–Follower Synchronization of Higher-Order Nonlinear Multi-Agent Systems via Fuzzy Neural Adaptive Sliding Control

**DOI:** 10.3390/s25247483

**Published:** 2025-12-09

**Authors:** Safeer Ullah, Muhammad Zeeshan Babar, Sultan Alghamdi, Ahmed S. Alsafran, Habib Kraiem, Abdullah A. Algethami

**Affiliations:** 1 Department of Electrical Engineering, Quaid-e-Azam College of Engineering & Technology, Sahiwal 57000, Pakistan; qurbandaysham@gmail.com; 2 School of Engineering and Physical Sciences, Heriot-Watt University, Edinburgh EH14 4AS, UK; 3 Department of Electrical and Computer Engineering, Faculty of Engineering, King Abdulaziz University, Jeddah 21589, Saudi Arabia; smalgamdi1@kau.edu.sa; 4 Center of Research Excellence in Renewable Energy and Power Systems, King Abdulaziz University, Jeddah 21589, Saudi Arabia; 5 Department of Electrical Engineering, College of Engineering, King Faisal University, Al-Ahsa 31982, Saudi Arabia; aalsafran@kfu.edu.sa; 6 Center for Scientific Research and Entrepreneurship, Northern Border University, Arar 73213, Saudi Arabia; alhabeeb.kareem@nbu.edu.sa; 7 Department of Mechanical Engineering, College of Engineering, Taif University, P.O. Box 11099, Taif 21944, Saudi Arabia; a_algethami@tu.edu.sa

**Keywords:** prescribed-time synchronization, multi-agent systems, fuzzy neural networks, non-singular terminal sliding mode control, adaptive robust control, leader–follower consensus

## Abstract

This paper introduces a novel control framework for prescribed-time synchronization of higher-order nonlinear multi-agent systems (MAS) subject to parametric uncertainties and external disturbances. The proposed method integrates a fuzzy neural network (FNN) with a robust non-singular terminal sliding mode controller (NTSMC) to ensure leader–follower consensus within a user-defined time horizon, regardless of the initial conditions. The FNN is employed to approximate unknown nonlinearities online, while an adaptive update law ensures accurate compensation for uncertainty. A terminal sliding manifold is designed to enforce finite-time convergence, and Lyapunov-based analysis rigorously proves prescribed-time stability and boundedness of all closed-loop signals. Simulation studies on a leader–follower MAS with four nonlinear agents under directed communication topology demonstrate the superiority of the proposed approach over conventional sliding mode control, achieving faster convergence, enhanced robustness, and improved adaptability against system uncertainties and external perturbations.

## 1. Introduction

Cooperative control of networked multi-agent systems (MAS) has attracted significant interest due to its broad applications in areas such as rendezvous coordination of nonholonomic agents [1], flocking-based altitude alignment [2], and formation control tasks [3,4]. In such systems, individual agents interact over structured communication topologies to achieve collective goals. A typical configuration is the leader–follower framework, where one agent (the leader) provides a reference trajectory, and the remaining agents (followers) synchronize their states accordingly. This framework facilitates controller implementation and reduces communication and computational overhead [5]. Although in real-world applications, not all followers may have direct access to the leader’s state or control input—especially in adversarial or resource-scarce situations. Therefore, contemporary distributed control approaches mostly consider the case where only a fraction of agents get partial leader information, which is relayed throughout the network [6]. This configuration adds complexity to the design of efficient and robust synchronization protocols with incomplete and uncertain information [7].

In the past ten years, substantial attention has been devoted to two core coordination problems in MASs, consensus and synchronization [8]. In consensus problems, all agents attempt to agree on a particular state variable, which is usually specified by its initial value, without depending on any leader agent, and this configuration is known as leaderless consensus [9]. Synchronization, however, deals with leader-follower problems where a leader agent produces a reference trajectory and the followers are required to track the leader along the path [10].

Although all these aspects have been theoretically investigated in great depth, satisfactory applications of them are still lacking. Practical MAS operate in dynamic and uncertain environments, in which unmodeled system dynamics, parameter variations, time-varying communication networks, and external noise may degrade performance or even cause instability [11]. Bridging such a gap to real applications, e.g., coordination of autonomous vehicles, clusters of robots, and sensor networks, requires control schemes that are adaptive to uncertainties and robust to time-varying configurations. In light of these difficulties, several control paradigms have been developed, including passivity-based methods [12], pinning control [13], energy-shaping methods [14], optimal control methods [15], and sliding mode control (SMC) methods.

Among the various SMC approaches, several notable variants have been developed to enhance robustness, adaptability, and precision in MAS coordination. Integral SMC [16] is commonly acknowledged for eliminating the reaching phase, thereby improving robustness and reducing the influence of initial conditions. Second-order SMC [17] introduces higher-order dynamics into the sliding variables, resulting in smoother control actions and reduced chattering compared to conventional SMC. Terminal SMC (TSMC) [18,19] ensures finite-time convergence with faster error reduction rates, making it ideal for applications that require rapid stabilization. Adaptive SMC [20,21] employs real-time tuning laws that allow the controller to adjust its gains online, effectively compensating for unknown disturbances and parameter variations without prior knowledge of uncertainty bounds. Overall, these SMC variants have proven to be powerful methods for achieving robust MAS coordination in the presence of severe nonlinearities, communication constraints, and external disturbances. Because they can guarantee finite-time or fixed-time convergence and mitigate chattering, these techniques have attracted significant attention in safety-critical and time-sensitive domains such as cooperative robotics, autonomous vehicle swarms, and distributed sensor networks.

Even considering the above advantages, finite-time conventional control methods (such as traditional TSMC) still exhibit some limitations. They are not entirely free from singularity in the control input and may suffer performance degradation due to sensitivity to initial conditions. Such drawbacks can restrict their applicability in systems that demand high precision and strict temporal performance. To overcome these issues, several advanced schemes have been introduced in the literature, including non-singular TSMC [22,23], trajectory transformation methods [24], redesigned sliding surfaces [25], and adaptive schemes incorporating saturation or fractional-order dynamics [26,27,28,29]. Although these methods appear to offer improved convergence and robustness characteristics, they generally lack a closed-form and exact expression for the settling time, which remains a critical limitation in time-sensitive applications.

Fixed-time control has been a promising choice, ensuring convergence within a predetermined upper limit that is entirely independent of the system’s initial state [30]. This feature is crucial for coordinating leader–follower synchronization with stringent timing requirements. Early studies explored fixed-time stability in nonlinear systems [31,32,33], while recent developments have extended these results to complex MAS architectures and high-order nonlinear dynamics. For example, Lei et al. proposed an event-triggered fixed-time stabilization framework for two-time-scale linear systems, ensuring practical fixed-time convergence under state-triggered sampling rules [34]. Similarly, Long et al. introduced a fixed-time consensus control scheme with prescribed performance and full-state constraints for nonlinear MAS, enhancing transient response and steady-state accuracy through adaptive event-triggered communication [35].

In parallel, Xiong et al. developed a model-free adaptive predictive control approach based on a broad-learning-system, which combines data-driven prediction with adaptive compensation to achieve robust consensus under denial-of-service attacks [36]. Likewise, reinforcement learning (RL) methods have recently been incorporated into fixed-time frameworks. Wang et al. presented a reinforcement-learning-based fixed-time prescribed performance consensus controller for stochastic nonlinear MAS with sensor faults, demonstrating strong robustness and convergence guarantees under probabilistic uncertainty [37]. Additionally, Ma et al. proposed a distributed fixed-time formation tracking control scheme using integral terminal SMC and disturbance observers for multi-wheeled mobile robots, validated experimentally under directed communication topologies [38]. In practical transportation systems, Liu and Xu designed a cooperative control framework for virtually coupled train formations that maintains safe inter-train spacing using barrier Lyapunov functions and adaptive control strategies [39].

Although these state-of-the-art methods have improved performance and reliability, most remain limited to linear or simplified nonlinear dynamics, struggling to maintain robustness under severe uncertainties and rapidly changing conditions. Moreover, excessive chattering and energy inefficiency persist in many fixed-time SMC formulations, particularly under high-gain or discontinuous control laws.

To overcome these challenges, this paper introduces a Neuro-Adaptive Prescribed-Time Non-Singular Terminal Sliding Mode Control (PNTSMC) framework for robust synchronization of higher-order uncertain nonlinear MAS. The proposed controller ensures convergence within a prescribed time for any initial condition while simultaneously reducing chattering and enhancing robustness. By integrating adaptive neural approximation into a non-singular terminal sliding mode framework, the learning capability is enhanced, allowing for compensation of unknown nonlinearities and achieving accurate tracking in the presence of matched uncertainties and external disturbances. This integrated topology offers a unified design for high-performance synchronization with guaranteed convergence time, making it suitable for implementation in complex and distributed intelligent systems.

The main contributions of this paper are summarized as follows:

- A novel non-singular terminal sliding surface is designed to ensure prescribed-time convergence in synchronization tasks, completely avoiding the singularity problem that commonly arises in conventional terminal SMC approaches.
- A fuzzy neural network (FNN)-based adaptive approximation mechanism is developed to estimate unknown nonlinear dynamics and external disturbances in real time. Compared with RBF-NNs or FLS-based methods, the proposed FNN offers faster learning, improved global approximation, and enhanced robustness through online parameter adaptation.
- A continuous reachability control law is formulated to effectively suppress chattering while preserving robustness, overcoming the high-frequency oscillation and actuator stress often observed in traditional SMC frameworks.
- Rigorous Lyapunov-based stability analysis is provided to guarantee prescribed-time synchronization and the boundedness of all closed-loop signals under matched uncertainties.
- Extensive simulations on a leader–follower network consisting of one leader and four followers verify that the proposed FNN-based PTSMC achieves faster convergence, higher robustness, and better adaptability than existing finite-time, fixed-time, and classical SMC schemes for uncertain nonlinear multi-agent systems.

The structure of this paper is outlined as follows. Section 2 provides the necessary mathematical background and an overview of graph theory, along with the formulation of the synchronization problem for higher-order MAS. The proposed PNTSMC scheme and its Lyapunov-based stability proof are presented in Section 3. Section 4 illustrates a simulation study validating the effectiveness of the proposed approach. Finally, Section 5 summarizes the work and highlights potential directions for future research.

## 2. Problem Formulation and Preliminaries

The growing application of multi-agent systems in contemporary engineering areas—ranging from robotics and autonomous vehicles to smart grids and sensor networks—has raised the need for robust, distributed control methods. The systems typically involve many agents communicating over intricate networks to pursue a common objective, for example, synchronization or formation tracking. One of the most challenging control problems in this regard is the leader-follower synchronization problem for higher-order uncertain nonlinear multi-agent systems, where each agent has complex dynamics and the system is affected by modeling uncertainties and communication constraints.

### 2.1. Challenges in Higher-Order Multi-Agent Systems

Multi-agent systems pose some particular challenges that require advanced control schemes:

1. Higher-Order Dynamics: In contrast to simple first- or second-order systems, most real-world agents have dynamics with more than a single integrator or nonlinear internal variables. The design of controllers for these types of systems requires a deep understanding of their own dynamics.
2. Nonlinearities: The agent dynamics are often nonlinear, making it inadequate to use the traditional linear control design techniques. Nonlinearities cause analytical complexity and restrict performance if not adequately addressed.
3. Uncertainties: Physical systems are always subject to unmodeled dynamics, disturbance inputs, and parametric uncertainties. These can cause a significant deterioration in stability and tracking performance.
4. Networked Interactions: Agents communicate via a communication structure given by a directed graph. The network topology, connectivity, and possibly delays have a significant impact on information flow and control performance.

### 2.2. Leader-Follower Synchronization Objective

In the leader–follower synchronization framework, a specific leader agent computes the reference trajectory, and the other follower agents are responsible for following it over time. The objective is to design distributed control inputs $u_{i}$ for each follower such that their states converge to those of the leader within a given time limit, regardless of the initial conditions. Such prescribed-time convergence is especially critical in time-sensitive applications, such as formations of uncrewed aerial vehicles and cooperative robotic systems.

Key aspects of the problem include:

1. Distributed Control: Each follower’s control input should be computed using its own state and information received only from neighboring agents, ensuring scalability and fault-tolerance.
2. prescribed-time Synchronization: In contrast to asymptotic or finite-time synchronization, prescribed-time synchronization guarantees uniform convergence time, which is particularly valuable in mission-critical scenarios.

### 2.3. Mathematical Modeling and Network Topology

The dynamics of each follower agent i∈{1,…,n} are described by:

(1) $$\begin{array}{cc} {\dot{x}}_{ij}= & x_{i,j+1},\;j=1,\ldots ,n-1;\\ \vdots \\ {\dot{x}}_{in}= & \varphi_{i}\left(x_{i}\right)+\gamma_{i}\left(x_{i}\right)u_{i}+\Delta_{i}\left(x_{i},t\right) \end{array}$$

where $x_{i}\in R^{n}$ is the state vector, $\varphi_{i}\left(\cdot \right)$ is a smooth nonlinear drift function, $\gamma_{i}\left(\cdot \right)>0$ is a smooth nonlinear input gain, $\Delta_{i}\left(\cdot ,\;\cdot \right)$ represents bounded uncertainty, and $u_{i}$ is the control input.

The leader’s dynamics are given by:

(2) $$\begin{array}{cc} {\dot{x}}_{0r}= & x_{0,r+1},\;\;\;\;r=1,\ldots ,n-1;\\ \vdots \\ {\dot{x}}_{0n}= & \varphi_{0}\left(t,x_{0}\right) \end{array}$$

with $x_{0}\in R^{n}$ representing the leader’s state and $\varphi_{0}\left(\cdot \right)$ being a continuous driving signal.

The communication topology is represented by a directed graph G=(V, E) , where the node set is $V=V_{0},\ldots ,V_{n}$ and the edge set satisfies E⊆V×V . An edge $(V_{j},\;V_{i})\in E$ signifies that agent *i* has access to the information transmitted by agent *j*. The weighted adjacency matrix is denoted by $A=[a_{ij}]$ , and the Laplacian matrix is defined as L=D−A , where *D* is the in-degree matrix. For the follower network, the matrices $\bar{A}$ and $\bar{L}$ represent the adjacency and Laplacian structures, respectively, while $\bar{B}$ characterizes the influence of the leader on the followers.

### 2.4. Synchronization Mismatch and Reformulated Objective

Let the synchronization error for the *i*-th agent be defined by:

(3) $$e_{i1}=x_{i1}-x_{01}$$

The synchronization error for the $k^{\mathrm{th}}$ state of agent *i* is:

(4) $$e_{ik}=\sum_{j=1}^{n}a_{ij}\left(x_{ik}-x_{jk}\right)+b_{i}\left(x_{ik}-x_{0k}\right),\;k=2,3,\ldots ,n$$

This captures both inter-follower discrepancies and the tracking error with respect to the leader. The dynamics of the mismatch vector $e_{i}={\left[e_{i1},\ldots ,e_{in}\right]}^{T}$ evolve as:  

(5) $$\begin{array}{cc} {\dot{e}}_{i1}= & e_{i2},\\ \vdots \\ {\dot{e}}_{in-1}= & e_{in},\\ {\dot{e}}_{in}= & \left(\sum_{j=1,j\neq i}^{n}a_{ij}+b_{i}\right)\left(\varphi_{i}\left(x_{i}\right)+\gamma_{i}\left(x_{i}\right)u_{i}\right)\\ & -\sum_{j=1,j\neq i}^{n}a_{ij}\left(\varphi_{j}\left(x_{j}\right)+\gamma_{j}\left(x_{j}\right)u_{j}\right)\\ & -b_{i}\varphi_{0}\left(t,x_{0}\right)+h_{i}\left(x,t\right) \end{array}$$

with $h_{i}\left(x,\;t\right)$ being the lumped uncertainty derived from all agents’ uncertainties.

(6) $$h_{i}\left(x,\;t\right)=\left(\sum_{j=1,j\neq i}^{n}a_{ij}+b_{i}\right)\Delta_{i}\left(x_{i},t\right)-\sum_{j=1,j\neq i}^{n}a_{ij}\Delta_{j}\left(x_{j},t\right)$$

where $\varphi_{i}\left(\cdot \right)$ , $\gamma_{i}\left(\cdot \right)$ are unknown nonlinearities, $h_{i}\left(x,t\right)$ represents lumped uncertainties, and $a_{ij}$ , $b_{i}$ are adjacency and pinning gains, respectively.

Thus, the control objective is reformulated as a prescribed-time regulation problem: Design a distributed control input $u_{i}$ for each follower such that $e_{ik}\to 0$ in fixed time despite unknown nonlinearities $\varphi_{i},\;\gamma_{i}$ and lumped uncertainty $h_{i}$ .

### 2.5. Assumptions

To make the problem tractable, the following assumptions are adopted:

- (A1) Controllability: $\gamma_{i}\left(x_{i}\right)>0$ for all $x_{i}$ , ensuring full actuation.
- (A2) Boundedness:
  1. Leader state: $∥x_{0}\;∥\leq \;\alpha_{M}$
  2. Leader dynamics: $|\varphi_{0}\left(t,\;x_{0}\right)|\leq |\chi \left(x_{0}\right)|$
  3. Uncertainties: $|\Delta_{i}\left(x_{i},\;t\right)|\leq C_{i}$
- (A3) Neighbor Awareness: Each follower has access to the states of its neighboring agents and, if directly connected, to the leader’s state.
- (A4) Smoothness: The nonlinear functions $f_{i}\left(\cdot \right)$ and $g_{i}\left(\cdot \right)$ are assumed to be at least $C^{1}$ -smooth with respect to their arguments. This guarantees the existence and continuity of their first-order derivatives, ensuring the validity of derivative-based operations in the control design and Lyapunov stability analysis.
- (A5) Leader–Follower Connectivity: The communication topology among agents is represented by a directed graph G=(V, E) that contains a directed spanning tree rooted at the leader. This ensures that at least one directed path exists from the leader to every follower agent, allowing the leader’s information to propagate throughout the network.

**Remark** **1.** 
*Assumption (A5) ensures that the leader’s information can propagate throughout the entire network, which is a standard and necessary condition for achieving consensus or synchronization in leader–follower MASs under directed topologies. Without this condition, certain followers may become isolated from the leader’s influence, making global synchronization impossible.*

### 2.6. Role of Graph Theory in Control Design

Graph-theoretic constructs such as adjacency matrices, Laplacians, and the leader influence matrix provide the foundation for designing distributed controllers and formulating the synchronization error. The existence of a directed spanning tree rooted at the leader ensures that the leader’s influence can propagate throughout the network, a necessary condition for achieving global synchronization.

### 2.7. Remark on State Availability

The proposed control framework assumes full state availability for all agents to compute mismatch variables. In practical applications, where higher-order states may not be directly measurable, observer-based techniques—such as high-gain observers or higher-order differentiators—can be employed to estimate the required states.

## 3. Control Design

This section introduces an adaptive control strategy based on fuzzy neural networks to realize prescribed-time synchronization in higher-order uncertain nonlinear multi-agent systems. The FNNs serve as universal approximators to estimate the unknown nonlinear components of the agent dynamics. A prescribed-time non-singular terminal sliding mode surface is designed to guarantee convergence within a fixed time regardless of the initial conditions, and adaptive weight update laws for the FNNs are derived using Lyapunov stability analysis.

### 3.1. Fuzzy Neural Network Approximation

**Figure 1:**
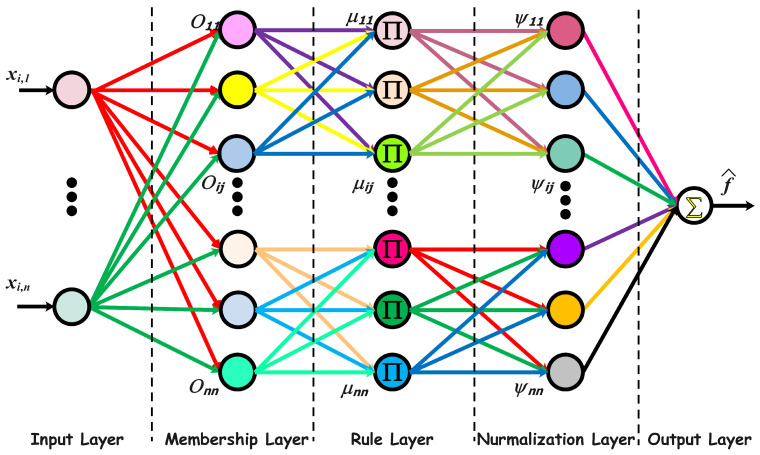
Architecture of the fuzzy neural network used for approximating the unknown nonlinear functions in the proposed FNN-PT-NTSMC framework.

To address the presence of unknown nonlinear functions in the agent dynamics, an FNN structure is employed for each agent *i*. This architecture, inspired by the Adaptive Neuro-Fuzzy Inference System (ANFIS), is designed to approximate the unknown smooth functions $\varphi_{i}\left(x_{i}\right)$ and $\gamma_{i}\left(x_{i}\right)$ , as illustrated in Figure 1.

The FNN operates in two primary phases:

1. Forward Propagation: The input vector $x_{i}$ is processed sequentially through the network’s five layers to produce an estimated output, $\hat{f}\left(x_{i},W\right)$ .
2. Parameter Adaptation: The network’s output weights, $W_{\varphi_{i}}$ and $W_{\gamma_{i}}$ , are updated online to minimize approximation errors. These updates are governed by adaptation laws derived from a Lyapunov-based stability analysis, ensuring system stability and prescribed-time convergence.

The FNN approximates the unknown functions as:

(7) $$\begin{array}{c} \varphi_{i}\left(x_{i}\right)\approx {\hat{\varphi }}_{i}\left(x_{i},W_{\varphi_{i}}\right)=W_{\varphi_{i}}^{T}{\bar{\Psi }}_{\varphi_{i}}\left(x_{i}\right)\\ \gamma_{i}\left(x_{i}\right)\approx {\hat{\gamma }}_{i}\left(x_{i},W_{\gamma_{i}}\right)=W_{\gamma_{i}}^{T}{\bar{\Psi }}_{\gamma_{i}}\left(x_{i}\right) \end{array}$$

where ${\bar{\Psi }}_{\varphi_{i}}\left(x_{i}\right)$ and ${\bar{\Psi }}_{\gamma_{i}}\left(x_{i}\right)$ are the normalized fuzzy basis function vectors, and $W_{\varphi_{i}}$ , $W_{\gamma_{i}}$ are the adaptive weight vectors.

The FNN consists of five computational layers:

Layer 1 (Input): This layer receives the agent’s state vector $x_{i}={\left[x_{i1},\ldots ,x_{in}\right]}^{T}$ .

(8) $$O_{1,j}=x_{ij},\;\;\;\;j=1,2,\ldots ,n$$

Layer 2 (Fuzzification): Each node uses a Gaussian membership function to determine the degree of membership for each input.

(9) $$\mu_{A_{jl}}\left(x_{ij}\right)=\exp\left(-\frac{{\left(x_{ij}-c_{jl}\right)}^{2}}{2\sigma_{jl}^{2}}\right)$$

The Gaussian membership function is chosen for the smoothness and differentiability. These characteristics enable stable calculations for adaptive parameter learning. Gaussian-shaped functions are preferred over triangular or trapezoidal functions, in which the points are nondifferentiable. The Gaussian shape provides smooth and continuous mappings. The learning process becomes more efficient, and the backpropagation of errors is also more effective. Its localized yet overlapping structure allows for better nonlinear approximations. The Gaussian function also achieves faster convergence and demonstrates lower sensitivity to parameter initialization. These make the Gaussian function particularly suited to fuzzy–neural adaptive control, where high precision and smooth adaptation are vital in the fuzzy–neural system to maintain control of stability and robustness for the system.

Layer 3 (Rule): This layer computes the firing strength of each fuzzy rule, typically using a product t-norm.

(10) $$\psi_{r}\left(x_{i}\right)=\prod_{j=1}^{n}\mu_{A_{rj}}\left(x_{ij}\right)$$

Layer 4 (Normalization): The firing strengths are normalized to ensure each rule has a proportional influence.

(11) $${\bar{\psi }}_{r}\left(x_{i}\right)=\frac{\psi_{r}\left(x_{i}\right)}{\sum_{k=1}^{N_{r}}\psi_{k}\left(x_{i}\right)}$$

This results in the fuzzy basis function vector:

(12) $$\bar{\Psi }\left(x_{i}\right)={\left[{\bar{\psi }}_{1}\left(x_{i}\right),\ldots ,{\bar{\psi }}_{N_{r}}\left(x_{i}\right)\right]}^{T}$$

Layer 5 (Output): For this layer, the TSK defuzzification technique is utilized to determine the final output.

(13) $$\hat{f}\left(x_{i},W\right)=\sum_{r=1}^{N_{r}}w_{r}{\bar{\psi }}_{r}\left(x_{i}\right)=W^{T}\bar{\Psi }\left(x_{i}\right)$$

We emphasize the TSK defuzzification method due to its analytical tractability and smooth fuzzy rule for controlling output mapping. TSK models, unlike Mamdani models, allow the use of linear, differentiable forms, which are necessary for Lyapunov-based stability control, parameter adaptation, and continuous-time fuzzy neural control.

Approximation errors are unavoidable; thus, we assume that optimal weight vectors exist, $W_{\varphi_{i}}^{*}$ and $W_{\gamma_{i}}^{*}$ , with possible approximations defined for the functions:

(14) $$\begin{array}{cc} \varphi_{i}\left(x_{i}\right)= & {W_{\varphi_{i}}^{*}}^{T}{\bar{\Psi }}_{\varphi_{i}}\left(x_{i}\right)+\epsilon_{\varphi_{i}}\left(x_{i}\right)\\ \gamma_{i}\left(x_{i}\right)= & {W_{\gamma_{i}}^{*}}^{T}{\bar{\Psi }}_{\gamma_{i}}\left(x_{i}\right)+\epsilon_{\gamma_{i}}\left(x_{i}\right) \end{array}$$

where $\epsilon_{\varphi_{i}}\left(x_{i}\right)$ and $\epsilon_{\gamma_{i}}\left(x_{i}\right)$ are the bounded approximation errors.

In the control structure, the FNN output is adaptively adjusted to force the sliding surface $s_{i}$ to zero. The adaptive laws update the output weights continuously with an instantaneous value of $s_{i}$ and normalized rule activations such that the approximation and tracking errors converge within the specified time.

**Remark** **2.** 
*The proposed FNN combines the interpretability of fuzzy logic with the adaptability of neural networks. Unlike RBF-NNs, which rely solely on local Gaussian activations, the FNN utilizes fuzzy membership rules for superior global approximation and enhanced resilience. Additionally, in contrast to conventional fuzzy logic systems, it eliminates the necessity for a priori rule base by allowing online adaptive adjustment of antecedent as well as consequent parameters, leading to quicker convergence and better adaptability in the presence of system uncertainties.*

### 3.2. Prescribed-Time Non-Singular Terminal Sliding Surface Design

For the convergence of tracking error in a pre-specified time, the sliding variable and associated control law are designed accordingly. Let T>0 be the pre-specified convergence time. From this condition, the sliding surface can be defined as:

(15) $$s_{i}=\sigma_{in}+\frac{c_{i}e_{in}}{{\left(T-t\right)}^{\delta }},\;\;c_{i}>0,\;\;0<\delta <1,\;\;T>0$$

where $\sigma_{in}$ is defined through the recursive NTSM as:

(16) $$\begin{array}{cc} \sigma_{i1} & =e_{i1}\\ \sigma_{ik} & =e_{ik}+\beta_{ik}\mathrm{sig}{\left(\sigma_{i,k-1}\right)}^{\alpha_{ik}},\;k=2,\ldots ,n \end{array}$$

with

(17) $$0<\alpha_{ik}<1,\;\beta_{ik}>0,\;\mathrm{sig}{\left(z\right)}^{\alpha }≜{\left|z\right|}^{\alpha }\mathrm{sign}\left(z\right)$$

**Remark** **3.** 
*This time-varying sliding surface injects an explicit time-decaying term that forces convergence by time t=T , i.e., the term $\frac{c_{i}e_{in}}{{\left(T-t\right)}^{\delta }}$ in* (15) *ensures exact convergence at t=T . In contrast, the use of $\mathrm{sig}{\left(z\right)}^{\alpha }$ in* (16) *avoids singularities. The sliding surface’s time-varying structure guarantees prescribed-time stability regardless of initial conditions.*

### 3.3. Prescribed-Time Control Law Design

Taking the time derivative of the sliding surface $s_{i}$ and expanding the terms gives:

(18) $${\dot{s}}_{i}={\dot{\sigma }}_{in}+\frac{c_{i}{\dot{e}}_{in}}{{\left(T-t\right)}^{\delta }}+\frac{c_{i}\delta e_{in}}{{\left(T-t\right)}^{\delta +1}}$$

With the recursive definition of $\sigma_{in}$ , its derivative is:

(19) $${\dot{\sigma }}_{in}={\dot{e}}_{in}+\beta_{in}\alpha_{in}\mathrm{sig}{\left(\sigma_{i,n-1}\right)}^{\alpha_{in}-1}{\dot{\sigma }}_{i,n-1}$$

Substituting (19) into (18), we get:  

(20) $$\begin{array}{cc} {\dot{s}}_{i}= & \left(1+\frac{c_{i}}{{\left(T-t\right)}^{\delta }}\right){\dot{e}}_{in}+\frac{c_{i}\delta e_{in}}{{\left(T-t\right)}^{\delta +1}}\\ \; & +\beta_{in}\alpha_{in}\mathrm{sig}{\left(\sigma_{i,n-1}\right)}^{\alpha_{in}-1}{\dot{\sigma }}_{i,n-1} \end{array}$$

The control input $u_{i}$ is then designed to ensure the stability of the system, driving the sliding surface to zero:

(21) $${\dot{s}}_{i}=-k_{i}s_{i}-\eta_{i}\mathrm{sgn}\left(s_{i}\right)$$

where $k_{i}>0$ , $\rho_{i}\in \left(0,1\right)$ , and $\eta_{i}>0$ are design constants. The control law $u_{i}$ compensates for uncertainties and enforces the sliding dynamics in (21):

(22) $$\begin{array}{cc} u_{i}= & {\left({\hat{\gamma }}_{i}\right)}^{-1}(-{\left(\sum_{j\neq i}a_{ij}+b_{i}\right)}^{-1}(\sum_{j\neq i}a_{ij}\left({\hat{\varphi }}_{j}+{\hat{\gamma }}_{j}u_{j}\right)+b_{i}\varphi_{0}\\ \; & -\left(\sum_{j\neq i}a_{ij}+b_{i}\right){\hat{\varphi }}_{i})-\beta_{in}\alpha_{in}\mathrm{sig}{\left(\sigma_{i,n-1}\right)}^{\alpha_{in}-1}{\dot{\sigma }}_{i,n-1}\\ \; & -\frac{c_{i}}{{\left(T-t\right)}^{\delta }}\left({\dot{e}}_{in}+\frac{\delta e_{in}}{T-t}\right)-k_{i}s_{i}-\eta_{i}\mathrm{sgn}\left(s_{i}\right)) \end{array}$$

**Remark** **4.** 
*This control law* (22) *ensures prescribed-time synchronization of each agent to the leader within time T, despite uncertainties, external disturbances, and inter-agent couplings. Neural network approximators can be updated online via adaptation laws to handle modeling errors.*

**Theorem** **1.** 
*Consider the nonlinear multi-agent system described in* (1)–(2)*, characterized by unknown but at least $C^{1}$ -smooth nonlinear functions $\varphi_{i}\left(x_{i}\right)$ and $\gamma_{i}\left(x_{i}\right)$ , and operating under Assumptions (A1)–(A5). If each follower agent employs the prescribed-time fuzzy neural adaptive non-singular terminal sliding mode control law defined by* (22)*, with the adaptive FNN weight update rules:*

(23) $$\begin{array}{cc} {\dot{\hat{W}}}_{\varphi_{i}} & =\Gamma_{\varphi_{i}}\left(s_{i}{\bar{\Psi }}_{\varphi_{i}}-\kappa_{\varphi_{i}}{\hat{W}}_{\varphi_{i}}\right),\\ {\dot{\hat{W}}}_{\gamma_{i}} & =\Gamma_{\gamma_{i}}\left(s_{i}{\bar{\Psi }}_{\gamma_{i}}u_{i}-\kappa_{\gamma_{i}}{\hat{W}}_{\gamma_{i}}\right), \end{array}$$

*and the sliding variable evolves according to*

$${\dot{s}}_{i}=-k_{i}s_{i}-\eta_{i}\;\mathrm{sgn}\left(s_{i}\right),$$

*Then the following properties hold:*

1. *All closed-loop signals, including ${\hat{W}}_{\varphi_{i}}$ , ${\hat{W}}_{\gamma_{i}}$ , and $u_{i}$ , remain bounded for all $t\in [t_{0},T)$ .*
2. *The synchronization errors $e_{i}\left(t\right)$ and sliding variables $s_{i}\left(t\right)$ converge to zero within the prescribed time T, i.e.,*
$$\lim_{t\to T^{-}}e_{i}\left(t\right)=0,\;\lim_{t\to T^{-}}s_{i}\left(t\right)=0,\;\forall i=1,2,\ldots ,N.$$

*This result holds provided the control parameters satisfy $c_{i}>0$ , 0<δ<1 , T>0 , $k_{i}>0$ , $0<\rho_{i}<1$ , and $\eta_{i}>0$ .*

**Proof.**  Consider the Lyapunov candidate for the whole network:

(24) $$V\left(t\right)=\sum_{i=1}^{N}V_{i}\left(t\right)=\sum_{i=1}^{N}\left(\frac{1}{2}s_{i}^{2}+\frac{1}{2\Gamma_{\varphi_{i}}}{\tilde{W}}_{\varphi_{i}}^{T}{\tilde{W}}_{\varphi_{i}}+\frac{1}{2\Gamma_{\gamma_{i}}}{\tilde{W}}_{\gamma_{i}}^{T}{\tilde{W}}_{\gamma_{i}}\right),$$

where ${\tilde{W}}_{\varphi_{i}}={\hat{W}}_{\varphi_{i}}-W_{\varphi_{i}}^{*}$ and ${\tilde{W}}_{\gamma_{i}}={\hat{W}}_{\gamma_{i}}-W_{\gamma_{i}}^{*}$ denote weight estimation errors.

Differentiate $V_{i}$ with respect to time to obtain  

(25) $${\dot{V}}_{i}=s_{i}{\dot{s}}_{i}+\frac{1}{\Gamma_{\varphi_{i}}}{\tilde{W}}_{\varphi_{i}}^{T}{\dot{\tilde{W}}}_{\varphi_{i}}+\frac{1}{\Gamma_{\gamma_{i}}}{\tilde{W}}_{\gamma_{i}}^{T}{\dot{\tilde{W}}}_{\gamma_{i}}.$$

Using the adaptive update laws (repeated here for clarity)

(26) $$\begin{array}{cc} {\dot{\hat{W}}}_{\varphi_{i}} & =\Gamma_{\varphi_{i}}\left(s_{i}{\bar{\Psi }}_{\varphi_{i}}-\kappa_{\varphi_{i}}{\hat{W}}_{\varphi_{i}}\right),\\ {\dot{\hat{W}}}_{\gamma_{i}} & =\Gamma_{\gamma_{i}}\left(s_{i}{\bar{\Psi }}_{\gamma_{i}}u_{i}-\kappa_{\gamma_{i}}{\hat{W}}_{\gamma_{i}}\right), \end{array}$$

and noting $\dot{\tilde{W}}=\dot{\hat{W}}$ , substitute into (25) to get

(27) $$\begin{array}{cc} {\dot{V}}_{i} & =s_{i}{\dot{s}}_{i}+{\tilde{W}}_{\varphi_{i}}^{T}\left(s_{i}{\bar{\Psi }}_{\varphi_{i}}-\kappa_{\varphi_{i}}{\hat{W}}_{\varphi_{i}}\right)+{\tilde{W}}_{\gamma_{i}}^{T}\left(s_{i}{\bar{\Psi }}_{\gamma_{i}}u_{i}-\kappa_{\gamma_{i}}{\hat{W}}_{\gamma_{i}}\right)\\ \; & =s_{i}{\dot{s}}_{i}+s_{i}{\tilde{W}}_{\varphi_{i}}^{T}{\bar{\Psi }}_{\varphi_{i}}+s_{i}{\tilde{W}}_{\gamma_{i}}^{T}{\bar{\Psi }}_{\gamma_{i}}u_{i}-\kappa_{\varphi_{i}}{\tilde{W}}_{\varphi_{i}}^{T}{\hat{W}}_{\varphi_{i}}-\kappa_{\gamma_{i}}{\tilde{W}}_{\gamma_{i}}^{T}{\hat{W}}_{\gamma_{i}}. \end{array}$$

From the control law and the augmented sliding construction, the full expression for ${\dot{s}}_{i}$ is

(28) $${\dot{s}}_{i}=-k_{i}s_{i}-\eta_{i}\mathrm{sgn}\left(s_{i}\right)-\frac{c_{i}}{{\left(T-t\right)}^{\delta }}\left({\dot{e}}_{in}+\frac{\delta e_{in}}{T-t}\right)+\Delta_{i},$$

where the lumped approximation/disturbance error is

(29) $$\Delta_{i}=\epsilon_{\varphi_{i}}\left(x_{i}\right)+\epsilon_{\gamma_{i}}\left(x_{i}\right)u_{i}+h_{i}\left(x,t\right),$$

and $h_{i}\left(x,t\right)$ collects the network-induced lumped uncertainties (as in the problem setup).

Substituting (28) into (27) yields

(30) $$\begin{array}{cc} {\dot{V}}_{i}\leq \;\; & -k_{i}s_{i}^{2}-\eta_{i}|s_{i}\left|+\right|s_{i}\left|\;\right|\Delta_{i}|\\ \; & +s_{i}{\tilde{W}}_{\varphi_{i}}^{T}{\bar{\Psi }}_{\varphi_{i}}+s_{i}{\tilde{W}}_{\gamma_{i}}^{T}{\bar{\Psi }}_{\gamma_{i}}u_{i}-\kappa_{\varphi_{i}}{\tilde{W}}_{\varphi_{i}}^{T}{\hat{W}}_{\varphi_{i}}-\kappa_{\gamma_{i}}{\tilde{W}}_{\gamma_{i}}^{T}{\hat{W}}_{\gamma_{i}}. \end{array}$$

Use the standard decomposition ${\tilde{W}}_{\varphi_{i}}^{T}{\bar{\Psi }}_{\varphi_{i}}={\hat{\varphi }}_{i}-\varphi_{i}+\epsilon_{\varphi_{i}}$ (and similarly for the γ -term) to group approximation terms. Define the aggregated bounded residual

(31) $${\bar{\Delta }}_{i}=\left(\sum_{j=1}^{N}a_{ij}+b_{i}\right)\left(\epsilon_{\varphi_{i}}+\epsilon_{\gamma_{i}}u_{i}\right)+h_{i}\left(x,t\right),$$

which satisfies $|{\bar{\Delta }}_{i}|\leq {\bar{\Delta }}_{i,\max}$ by assumption.

Choose the switching gain $\eta_{i}$ to satisfy the sufficient condition

(32) $$\eta_{i}\geq \left|{\bar{\Delta }}_{i}\right|,$$

so that the switching action dominates the uncertain terms. With this choice and noting $-\kappa {\tilde{W}}^{T}\hat{W}\leq 0$ , (30) reduces to the conservative bound

(33) $${\dot{V}}_{i}\leq -k_{i}s_{i}^{2}.$$

Hence $\dot{V}\left(t\right)=\sum_{i}{\dot{V}}_{i}\leq -\sum_{i}k_{i}s_{i}^{2}\leq 0$ , and V(t) is nonincreasing and bounded on $t\in [t_{0},T)$ . This establishes boundedness of all closed-loop signals (weights, control inputs, states) for t<T .

To obtain the explicit decay rate that yields prescribed-time convergence, we exploit the augmented sliding structure. On the sliding manifold (or during the reaching phase under the designed reaching law), the time-varying term with ${\left(T-t\right)}^{-\delta }$ enforces an accelerated decay. In particular, one can show (by using the sliding-surface definition and the NTSM recursion) that there exists a positive constant $\mu_{i}>0$ such that for $t\in [t_{0},T)$
  

(34) $$-k_{i}s_{i}^{2}\leq -\frac{2\delta }{T-t}V_{i}\left(t\right).$$

Here the constant $\mu_{i}$ and parameter choices are determined by the NTSM gains $c_{i},\;\beta_{in},\;\alpha_{in}$ and the network weights; details follow standard prescribed-time NTSM derivations—(see e.g., [40]).

Combining (34) across agents yields the differential inequality

(35) $$\dot{V}\left(t\right)\leq -\frac{2\delta }{T-t}V\left(t\right),\;t\in \left[t_{0},T\right).$$

Solving (35) by separation of variables gives

(36) $$V\left(t\right)\leq V\left(t_{0}\right){\left(\frac{T-t}{T-t_{0}}\right)}^{2\delta },\;\;\;t\in \left[t_{0},T\right),$$

which implies $\lim_{t\to T^{-}}V\left(t\right)=0$ . Since V(t) is positive definite in the tracking variables, we obtain

$$\lim_{t\to T^{-}}s_{i}\left(t\right)=0,\;\;\lim_{t\to T^{-}}e_{i}\left(t\right)=0,\;\forall i.$$

**Remark** **5.** *Theorem 1 establishes prescribed-time convergence on $t\in [t_{0},T)$ , with guaranteed limit $\lim_{t\to T^{-}}e_{i}\left(t\right)=0$ . For numerical robustness, the singular term ${\left(T-t\right)}^{-\delta }$ used in the control law is regularized as ${\left(T-t+\varepsilon \right)}^{-\delta }$ with a small ε>0 (e.g., $\varepsilon =10^{-3}$ ), ensuring smooth control action and eliminating division-by-zero near t=T .*

Therefore, all closed-loop signals remain bounded for $t\in [t_{0},T)$ and the synchronization errors converge to zero at the prescribed time in the limiting sense $t\to T^{-}$ , completing the proof.    □

### 3.4. Prescribed-Time Convergence

To verify the prescribed-time convergence capability of the proposed control method, we conduct a detailed analysis that demonstrates the tracking errors $e_{i}$ and the sliding variables $s_{i}$ reach zero precisely at the predefined time *T*, regardless of the initial system states. Consider the sliding surface dynamics:

(37) $${\dot{s}}_{i}=-k_{i}s_{i}-\eta_{i}\;\mathrm{sgn}\left(s_{i}\right)$$

This represents a finite-time stable system. Therefore, the sliding variable $s_{i}$ converges to zero in a finite time $t_{s}\leq T$ . However, the constructed time-varying sliding surface includes a singular term:

(38) $$s_{i}=\sigma_{in}+\frac{c_{i}e_{in}}{{\left(T-t\right)}^{\delta }}$$

**Lemma** **1.** 
*The sliding surface $s_{i}$ converges to zero in prescribed time T, i.e., $s_{i}\left(t\right)=0$ for all t≥T .*

**Proof.**  From the structure of $s_{i}$ in (38), as $t\to T^{-}$ , the term $\frac{c_{i}e_{in}}{{\left(T-t\right)}^{\delta }}$ must remain finite (since $s_{i}$ is bounded). This requires

(39) $$e_{in}\left(t\right)=O\left({\left(T-t\right)}^{\delta }\right)$$

and thus $e_{in}\left(t\right)\to 0$ as $t\to T^{-}$ . The recursive structure of $\sigma_{in}$ then ensures all tracking errors $e_{i1},...,e_{in}$ converge to zero by time *T*.    □

On the sliding surface $s_{i}=0$ , we have:   

(40) $$\sigma_{in}=-\frac{c_{i}e_{in}}{{\left(T-t\right)}^{\delta }}$$

Taking the time derivative yields:

(41) $${\dot{\sigma }}_{in}=-\frac{c_{i}{\dot{e}}_{in}}{{\left(T-t\right)}^{\delta }}-\frac{c_{i}\delta e_{in}}{{\left(T-t\right)}^{\delta +1}}$$

Recall the recursive NTSM structure:

(42) $$\sigma_{in}=e_{in}+\beta_{in}\;\mathrm{sig}{\left(\sigma_{i,n-1}\right)}^{\alpha_{in}}$$

This implies that:

(43) $$e_{in}=-\beta_{in}\;\mathrm{sig}{\left(\sigma_{i,n-1}\right)}^{\alpha_{in}}-\frac{c_{i}e_{in}}{{\left(T-t\right)}^{\delta }}$$

From (43), it is evident that $e_{in}$ must decay faster than ${\left(T-t\right)}^{\delta }$ to avoid the singularity at t=T , ensuring convergence of both $s_{i}$ and $e_{in}$ .

Lyapunov-Based Prescribed-Time Stability Analysis: To formally verify convergence, consider the Lyapunov candidate:

(44) $$V_{e}=\frac{1}{2}e_{in}^{2}$$

Differentiating (44) with respect to time:

(45) $${\dot{V}}_{e}=e_{in}{\dot{e}}_{in}$$

From the sliding condition $s_{i}=0$ , substitute for ${\dot{e}}_{in}$ :

(46) $${\dot{e}}_{in}=-\frac{{\left(T-t\right)}^{\delta }}{c_{i}}\left({\dot{\sigma }}_{in}+\frac{c_{i}\delta e_{in}}{{\left(T-t\right)}^{\delta +1}}\right)$$

Substituting (46) into (45):

(47) $${\dot{V}}_{e}=-\frac{{\left(T-t\right)}^{\delta }}{c_{i}}e_{in}{\dot{\sigma }}_{in}-\frac{\delta }{T-t}e_{in}^{2}$$

Since ${\dot{\sigma }}_{in}$ is bounded (due to bounded dynamics and control design), the dominant term is:

(48) $${\dot{V}}_{e}\leq -\frac{\delta }{T-t}e_{in}^{2}=-2\frac{\delta }{T-t}V_{e}$$

This yields a time-varying differential inequality. Solving it gives:

(49) $$V_{e}\left(t\right)\leq V_{e}\left(t_{0}\right){\left(\frac{T-t}{T-t_{0}}\right)}^{2\delta }$$

Therefore:

(50) $$|e_{in}\left(t\right)|\leq |e_{in}\left(t_{0}\right)|{\left(\frac{T-t}{T-t_{0}}\right)}^{\delta }$$

As t→T , this implies:

(51) $$\lim_{t\to T}e_{in}\left(t\right)=0$$

demonstrating prescribed-time convergence of the tracking error $e_{in}$ . Given inequality (50), it is clear that:

(52) $$\lim_{t\to T}\left|e_{in}\left(t\right)\right|=0$$

Thus, each agent’s tracking error converges to zero exactly at the predefined time *T*, regardless of initial conditions, confirming prescribed-time synchronization.

**Remark** **6.** 
*The convergence time T is explicitly imposed by the designer and is independent of the initial conditions of the agents. This is a stronger guarantee than finite-time or fixed-time convergence and is particularly valuable for time-critical cooperative control applications.*

The proposed control framework, summarized in Algorithm 1, outlines the step-by-step implementation of the designed strategy.
**Algorithm 1** Fuzzy Neural Network-based PNTSMC

**Require:** 
System parameters for each agent i=1,…,N : T>0 , 0<δ<1 , $c_{i}>0$ , $k_{i}>0$ , $\eta_{i}>0$ , $\beta_{ik},\alpha_{ik}$ , adaptation gains $\Gamma_{\varphi_{i}},\Gamma_{\gamma_{i}}$ , leakage $\kappa_{\varphi_{i}},\kappa_{\gamma_{i}}$ , sampling time Δt , adjacency $a_{ij}$ , leader coupling $b_{i}$ .1:Initialize $t\leftarrow t_{0}$ , weights ${\hat{W}}_{\varphi_{i}}\left(t_{0}\right)$ , ${\hat{W}}_{\gamma_{i}}\left(t_{0}\right)$ , states $x_{i}\left(t_{0}\right)$ and control $u_{i}\left(t_{0}\right)$ .2:**while** t<T **do**3:    **for** each follower agent i=1,…,N **do**4:        **Measure / estimate** current state $x_{i}\left(t\right)$ and neighbors’ states (if needed).5:        **Compute tracking errors** $e_{i1},\ldots ,e_{in}$ (e.g., $e_{ij}=x_{ij}-x_{0j}$ ) and form $e_{in}$ .6:        **Compute recursive NTSM terms**:
   
$\sigma_{i1}\leftarrow e_{i1}$

7:        **for** k=2 to *n* **do**
   
$\sigma_{ik}\leftarrow e_{ik}+\beta_{ik}\;\mathrm{sig}{\left(\sigma_{i,k-1}\right)}^{\alpha_{ik}}$

8:        **end for**9:        **Form sliding variable**
   
$s_{i}\leftarrow \sigma_{in}+\frac{c_{i}e_{in}}{{\left(T-t\right)}^{\delta }}$

10:        **FNN forward pass:** evaluate normalized basis vectors ${\bar{\Psi }}_{\varphi_{i}}\left(x_{i}\right),\;{\bar{\Psi }}_{\gamma_{i}}\left(x_{i}\right)$ (layers 1–4)
   
${\hat{\varphi }}_{i}\leftarrow {\hat{W}}_{\varphi_{i}}^{T}{\bar{\Psi }}_{\varphi_{i}}\left(x_{i}\right)$ , ${\hat{\gamma }}_{i}\leftarrow {\hat{W}}_{\gamma_{i}}^{T}{\bar{\Psi }}_{\gamma_{i}}\left(x_{i}\right)$

11:        **Compute derivative terms (approx.)** ${\dot{e}}_{in},\;{\dot{\sigma }}_{i,n-1}$ (use numerical differentiation or observer).12:        **Compute control input** using (22) (discrete-time approximation):
$$\begin{array}{c} u_{i}\leftarrow {\left({\hat{\gamma }}_{i}\right)}^{-1}(-{\left(\sum_{j\neq i}a_{ij}+b_{i}\right)}^{-1}(\sum_{j\neq i}a_{ij}\left({\hat{\varphi }}_{j}+{\hat{\gamma }}_{j}u_{j}\right)\;\\ \;\;+b_{i}\varphi_{0}-\left(\sum_{j\neq i}a_{ij}+b_{i}\right){\hat{\varphi }}_{i})-\beta_{in}\alpha_{in}\mathrm{sig}{\left(\sigma_{i,n-1}\right)}^{\alpha_{in}-1}{\dot{\sigma }}_{i,n-1}\\ \;\;-\frac{c_{i}}{{\left(T-t\right)}^{\delta }}\left({\dot{e}}_{in}+\frac{\delta e_{in}}{T-t}\right)-k_{i}s_{i}-\eta_{i}\mathrm{sgn}\left(s_{i}\right)) \end{array}$$
13:        **Apply control** $u_{i}$ to the plant (hold for Δt ).14:        **Weight adaptation (online)**: update FNN weights using (23) (Euler step)
$$\begin{array}{c} {\hat{W}}_{\varphi_{i}}\leftarrow {\hat{W}}_{\varphi_{i}}+\Delta t\;\Gamma_{\varphi_{i}}\left(s_{i}{\bar{\Psi }}_{\varphi_{i}}-\kappa_{\varphi_{i}}{\hat{W}}_{\varphi_{i}}\right),\\ {\hat{W}}_{\gamma_{i}}\leftarrow {\hat{W}}_{\gamma_{i}}+\Delta t\;\Gamma_{\gamma_{i}}\left(s_{i}{\bar{\Psi }}_{\gamma_{i}}u_{i}-\kappa_{\gamma_{i}}{\hat{W}}_{\gamma_{i}}\right). \end{array}$$
15:        Optional: enforce projection or bounding on ${\hat{W}}_{\varphi_{i}},{\hat{W}}_{\gamma_{i}}$ to keep them in a compact set.16:    **end for**17:    Advance time: t←t+Δt
18:**end while**19:**Post-time behavior** ( t≥T ): set $s_{i}=0$ , $e_{ij}=0$ for all agents (the idealized result). Optionally switch to a steady-state controller or hold the last $u_{i}$ .
**Ensure:** 
Prescribed-time synchronization $e_{i}\left(t\right)\to 0$ and $s_{i}\left(t\right)\to 0$ for all $t\in [t_{0},T)$ (under the assumptions and parameter choices in the theorem).


## 4. Illustrative Example: Synchronization Control of a Networked System

**Figure 2:**
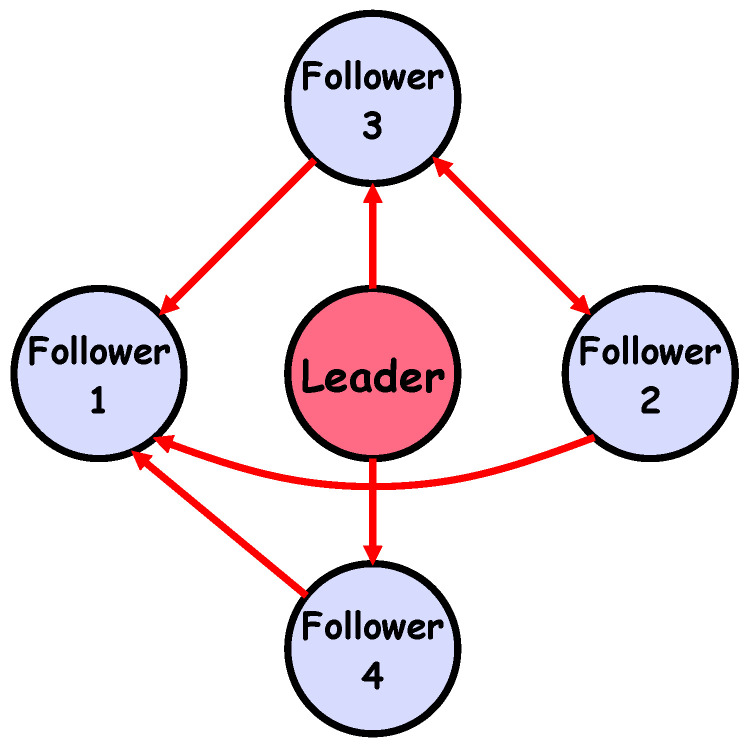
Configuration of the network consisting of one leader and four follower agents under a defined topology.

To demonstrate the effectiveness of the proposed synchronization control approach for networked agents, a simulation is conducted on a system comprising one leader and four follower agents operating under uncertain disturbances. The communication topology, shown in Figure 2, represents a directed graph that illustrates the information flow among the agents.

In this illustrative scenario, both the leader and the followers are modeled as second-order dynamical systems. The main objective is to demonstrate the controller’s capability in ensuring that all follower agents asymptotically track the leader’s trajectory, even in the presence of external matched disturbances acting on the followers. The subsequent subsections describe the agent dynamics, graph-theoretic properties of the network, and synchronization mismatch variables.

### 4.1. System Dynamics of the Leader and Followers

The following set of first-order differential equations describes the leader’s dynamics:

(53) $$\begin{array}{cc} {\dot{x}}_{01} & =x_{02},\\ {\dot{x}}_{02} & =\varphi_{0}\left(x_{01},x_{02},t\right), \end{array}$$

where $\varphi_{0}\left(x_{01},x_{02},t\right)=-\frac{\sin(x_{01})}{1+\exp(-t)}$ represents a time-varying exogenous input. This formulation ensures that $\varphi_{0}\left(0,0,t\right)=0$ , guaranteeing equilibrium at the origin in the absence of external excitation.

Each $i^{\mathrm{th}}$ follower agent ( i=1,2,3,4 ) is governed by the nonlinear dynamics:

(54) $$\begin{array}{cc} {\dot{x}}_{i1} & =x_{i2},\\ {\dot{x}}_{i2} & =\varphi_{i}\left(x_{i}\right)+\gamma_{i}\left(x_{i}\right)u_{i}+\Delta_{i}\left(x_{i},t\right), \end{array}$$

where $x_{i}={\left[x_{i1},x_{i2}\right]}^{T}$ is the state vector, $\Delta_{i}\left(x_{i},t\right)=0.5\sin\left(t\right)$ denotes a matched-type bounded disturbance, and $\varphi_{i}\left(x_{i}\right)=-\frac{g}{l}\sin\left(x_{i1}\right)$ represents pendulum-like dynamics with gravity *g* and length *l*. The control gain is given by $\gamma_{i}\left(x_{i}\right)=\frac{1}{ml^{2}}$ , where *m* is the pendulum mass. The goal is to design $u_{i}$ such that $x_{i}\left(t\right)$ asymptotically tracks $x_{0}\left(t\right)$ despite disturbances.

### 4.2. Network Topology and Interconnection Matrices

The inter-agent communication is described by a directed graph shown in Figure 2. The graph structure is captured by the adjacency matrix $A\in R^{5\times 5}$ (including the leader as node 1), the Laplacian matrix $\bar{L}\in R^{4\times 4}$ for the follower subgraph, and the leader influence matrix $\bar{B}\in R^{4\times 4}$ :

(55) $$\begin{array}{c} A=\left[\begin{array}{ccccc} 0 & 0 & 0 & 0 & 0\\ 0 & 0 & 1 & 1 & 1\\ 0 & 0 & 0 & 1 & 0\\ 1 & 0 & 1 & 0 & 0\\ 1 & 0 & 0 & 0 & 0 \end{array}\right],\bar{L}=\left[\begin{array}{cccc} 3 & -1 & -1 & -1\\ 0 & 1 & -1 & 0\\ 0 & -1 & 1 & 0\\ 0 & 0 & 0 & 0 \end{array}\right]\\ \bar{B}=\mathrm{diag}\left[0,0,1,1\right]. \end{array}$$

where $A_{ij}=1$ denotes a directed link from agent *j* to agent *i*, and zero otherwise. $\bar{L}$ captures the internal connectivity of follower agents, while $\bar{B}$ indicates which followers directly receive information from the leader.

### 4.3. Control Design for the Illustrative Example

Based on the provided illustrative example, we now detail the design of the fuzzy neural network-based adaptive prescribed-time control technique for the networked system. The system comprises one leader and four followers, each with second-order dynamics.

#### 4.3.1. Synchronization Error Dynamics

The synchronization mismatch variables for each follower agent i∈{1,…,4} are defined as:

(56) $$\begin{array}{cc} e_{i1} & =\sum_{j=1}^{4}a_{ij}\left(x_{i1}-x_{j1}\right)+b_{i}\left(x_{i1}-x_{01}\right)\\ e_{i2} & =\sum_{j=1}^{4}a_{ij}\left(x_{i2}-x_{j2}\right)+b_{i}\left(x_{i2}-x_{02}\right) \end{array}$$

These represent weighted state deviations from neighboring agents and the leader, based on the network structure. The controller aims to drive $e_{i1},\;e_{i2}\to 0$ as t→T .

The dynamics of these error variables are derived by taking their time derivatives:

(57) $$\begin{array}{cc} {\dot{e}}_{i1}= & \sum_{j=1}^{4}a_{ij}\left({\dot{x}}_{i1}-{\dot{x}}_{j1}\right)+b_{i}\left({\dot{x}}_{i1}-{\dot{x}}_{01}\right)\\ = & \sum_{j=1}^{4}a_{ij}\left(x_{i2}-x_{j2}\right)+b_{i}\left(x_{i2}-x_{02}\right)=e_{i2} \end{array}$$

(58) $$\begin{array}{cc} {\dot{e}}_{i2} & =\sum_{j=1}^{4}a_{ij}\left({\dot{x}}_{i2}-{\dot{x}}_{j2}\right)+b_{i}\left({\dot{x}}_{i2}-{\dot{x}}_{02}\right)\\ \; & =\left(\sum_{j=1}^{4}a_{ij}+b_{i}\right){\dot{x}}_{i2}-\sum_{j=1}^{4}a_{ij}{\dot{x}}_{j2}-b_{i}{\dot{x}}_{02}\\ \; & =\left(\sum_{j=1}^{4}a_{ij}+b_{i}\right)\left(\varphi_{i}+\gamma_{i}u_{i}+\Delta_{i}\right)-\sum_{j=1}^{4}a_{ij}\left(\varphi_{j}+\gamma_{j}u_{j}+\Delta_{j}\right)-b_{i}\varphi_{0} \end{array}$$

Let $N_{i}=\left\{j\in \left\{1,\ldots ,4\right\}|a_{ij}>0\right\}\cup \left\{0|b_{i}>0\right\}$ denote the set of neighbors of agent *i*, including the leader. The control input $u_{i}$ is designed to drive $e_{i1}$ and $e_{i2}$ to zero within a prescribed time *T*.

#### 4.3.2. Prescribed-Time Non-Singular Terminal Sliding Surface

For the second-order system, a time-varying sliding surface is formulated to guarantee prescribed-time convergence. The objective is to ensure that all tracking errors vanish exactly at the predefined time *T*, regardless of their initial values. The sliding variable $s_{i}$ is defined as:

(59) $$s_{i}=e_{i2}+\beta_{i}\mathrm{sig}{\left(e_{i1}\right)}^{\alpha_{i}}+\frac{c_{i}e_{i1}}{{\left(T-t\right)}^{\delta }}$$

where $\beta_{i}>0$ , $0<\alpha_{i}<1$ , $c_{i}>0$ , and 0<δ<1 are positive design constants.

#### 4.3.3. FNN-Based Adaptive Control Law Design

The control law $u_{i}$ is designed to satisfy the sliding condition ${\dot{s}}_{i}=0$ (or a more general stable form ${\dot{s}}_{i}=-k_{i}s_{i}-\eta_{i}\mathrm{sgn}\left(s_{i}\right)$ for robustness). Taking the time derivative of $s_{i}$ :

(60) $${\dot{s}}_{i}={\dot{e}}_{i2}+\beta_{i}\alpha_{i}\mathrm{sig}{\left(e_{i1}\right)}^{\alpha_{i}-1}{\dot{e}}_{i1}+\frac{c_{i}{\dot{e}}_{i1}}{{\left(T-t\right)}^{\delta }}+\frac{c_{i}\delta e_{i1}}{{\left(T-t\right)}^{\delta +1}}$$

Using ${\dot{e}}_{i1}=e_{i2}$ and substituting the expression for ${\dot{e}}_{i2}$ from (58) into (60):

(61) $$\begin{array}{cc} {\dot{s}}_{i}= & \left(\sum_{j=1}^{4}a_{ij}+b_{i}\right)\left(\varphi_{i}+\gamma_{i}u_{i}+\Delta_{i}\right)-\sum_{j=1}^{4}a_{ij}\left(\varphi_{j}+\gamma_{j}u_{j}+\Delta_{j}\right)-b_{i}\varphi_{0}\\ \; & +\beta_{i}\alpha_{i}\mathrm{sig}{\left(e_{i1}\right)}^{\alpha_{i}-1}e_{i2}+\frac{c_{i}e_{i2}}{{\left(T-t\right)}^{\delta }}+\frac{c_{i}\delta e_{i1}}{{\left(T-t\right)}^{\delta +1}} \end{array}$$

The unknown nonlinear functions $\varphi_{i},\;\gamma_{i}$ and disturbances $\Delta_{i}$ are approximated by FNNs. The FNN approximations are denoted by ${\hat{\varphi }}_{i}={\hat{W}}_{\varphi_{i}}^{T}{\bar{\Psi }}_{\varphi_{i}}\left(x_{i}\right)$ and ${\hat{\gamma }}_{i}={\hat{W}}_{\gamma_{i}}^{T}{\bar{\Psi }}_{\gamma_{i}}\left(x_{i}\right)$ . To ensure stability and convergence, the control law is designed to cancel the nonlinearities and disturbances. The resulting control law $u_{i}$ is given by:

(62) $$\begin{array}{cc} u_{i}= & {\hat{\gamma }}_{i}^{-1}(-{\hat{\varphi }}_{i}-{\left(\sum_{j=1}^{4}a_{ij}+b_{i}\right)}^{-1}\left(-\sum_{j=1}^{4}a_{ij}\left({\hat{\varphi }}_{j}+{\hat{\gamma }}_{j}u_{j}\right)-b_{i}\varphi_{0}\right)\\ \; & -{\left(\sum_{j=1}^{4}a_{ij}+b_{i}\right)}^{-1}\left(\beta_{i}\alpha_{i}\mathrm{sig}{\left(e_{i1}\right)}^{\alpha_{i}-1}e_{i2}+\frac{c_{i}e_{i2}}{{\left(T-t\right)}^{\delta }}+\frac{c_{i}\delta e_{i1}}{{\left(T-t\right)}^{\delta +1}}\right)\\ \; & -{\left(\sum_{j=1}^{4}a_{ij}+b_{i}\right)}^{-1}\left(k_{i}s_{i}+\eta_{i}\mathrm{sgn}\left(s_{i}\right)\right)) \end{array}$$

where $k_{i}>0$ and $\eta_{i}>0$ are the control gains for robustness and stability.

#### 4.3.4. FNN Adaptive Laws

The weights of the FNNs are updated online using the following adaptive laws, which are derived to guarantee the boundedness of the weight estimation errors:

(63) $$\begin{array}{cc} {\dot{\hat{W}}}_{\varphi_{i}}= & \Gamma_{\varphi_{i}}\left(s_{i}{\bar{\Psi }}_{\varphi_{i}}\left(x_{i}\right)\left(\sum_{j=1}^{4}a_{ij}+b_{i}\right)-\kappa_{\varphi_{i}}{\hat{W}}_{\varphi_{i}}\right)\\ {\dot{\hat{W}}}_{\gamma_{i}}= & \Gamma_{\gamma_{i}}\left(s_{i}{\bar{\Psi }}_{\gamma_{i}}\left(x_{i}\right)u_{i}\left(\sum_{j=1}^{4}a_{ij}+b_{i}\right)-\kappa_{\gamma_{i}}{\hat{W}}_{\gamma_{i}}\right) \end{array}$$

where $\Gamma_{\varphi_{i}},\Gamma_{\gamma_{i}}$ are positive definite learning rate matrices and $\kappa_{\varphi_{i}},\kappa_{\gamma_{i}}$ are positive design constants. These laws ensure that the approximation errors are compensated for, allowing the system to achieve prescribed-time synchronization.

#### 4.3.5. Theoretical Guarantee

**Theorem** **2.** 
*Consider the leader-follower network described above. Suppose each follower’s control input $u_{i}$ is designed as per the control law* (62)*, and the FNN weights are updated via the adaptation laws* (63)*. Then, for properly selected design parameters $c_{i}>0,\;\delta \in \left(0,\;1\right),\;T>0,\;k_{i}>0,\;\eta_{i}>0$ , the synchronization errors $e_{i1},\;e_{i2}$ and sliding surfaces $s_{i}$ converge to zero within the prescribed time T, i.e.,*

(64) $$e_{i1}\left(t\right),\;e_{i2}\left(t\right),\;s_{i}\left(t\right)\to 0\;as\;t\to T^{-},\;\;\;\;\;\forall i\in \left\{1,2,3,4\right\}.$$

*This implies that all follower agents synchronize with the leader within a guaranteed finite time, despite the presence of modeling uncertainties, nonlinear dynamics, and inter-agent couplings.*

### 4.4. Simulation Results

This section presents a detailed simulation analysis aimed at assessing the performance and robustness of the new control scheme, particularly in the presence of matched-type, time-varying disturbances. Additionally, comparative performance evaluation is conducted with respect to the conventional SMC approach to identify the improvements achieved by the new method.

#### 4.4.1. Controller Parameters

The control parameters used in the simulations are listed in Table 1. They play a crucial role in shaping the closed-loop dynamics of the multi-agent system and in adjusting the responsiveness of the control strategy.

#### 4.4.2. Simulation Setup

The considered system is a five-agent system, where one agent, i=1 , is designated as the leader, while the other agents, i=2,…,5 , are followers. Its communication network is formed according to Figure 2. The agents are initialized with unique values for their initial states. Simulation results are obtained using MATLAB/Simulink (version R2024b) with an Euler integration (One-Step) scheme and a step size of 0.01 s. For robustness evaluation, the followers are subjected to sinusoidal time-varying disturbances during simulation.

#### 4.4.3. Tracking Performance

**Figure 3:**
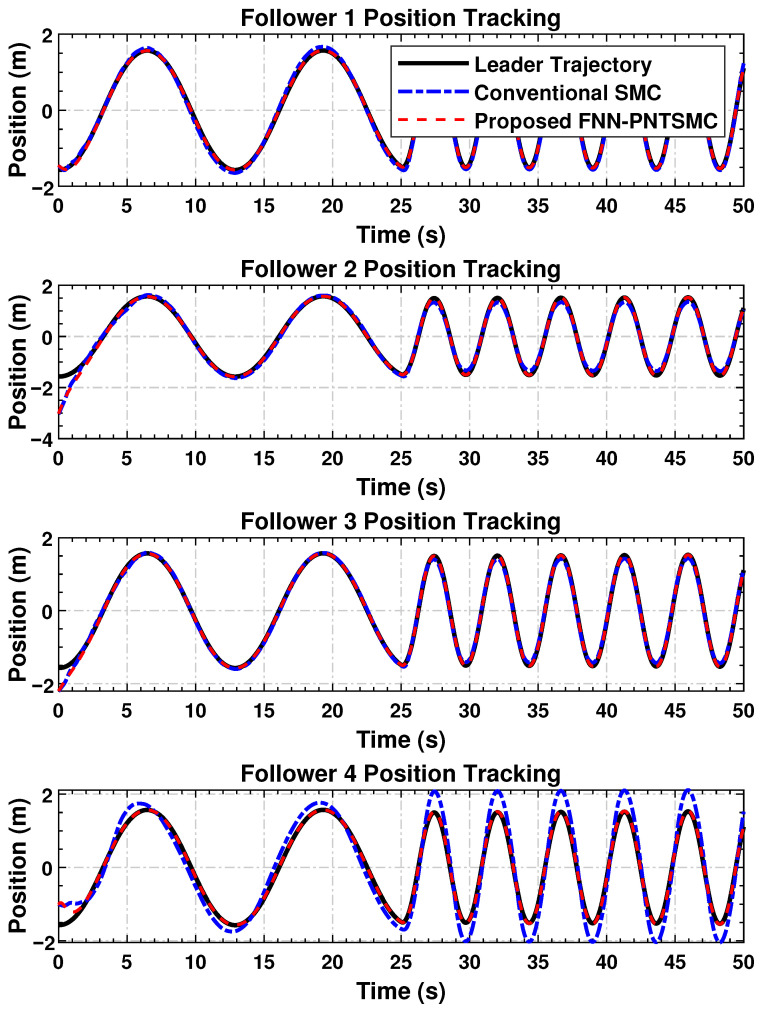
The four follower agents’ position trajectories converging to the path of the leader under the designed control strategy.

The positional convergence pattern of the follower agents with respect to the leader is shown in Figure 3. The tracking accuracy of the proposed control law, despite external perturbations, further affirms the results of the previous section. The results of the proposed framework demonstrate a significant improvement over classical SMC, characterized by smoother transitions and lower residual errors.

As shown, all agents effectively and efficiently converge to the leader’s trajectory, even in the presence of the prescribed time constraints and disturbances. The active control achieves minimal overshoot and oscillation, further demonstrating the responsive and well-tuned nature of the proposed control system. In contrast, while the conventional SMC-based controller exhibited some steady-state error and noticeable chattering, the proposed controller reached a steady state with virtually no error. The residual chattering and steady-state deviations in the SMC case could pose challenges in precision or safety-critical systems.

#### 4.4.4. Mismatch Convergence

**Figure 4:**
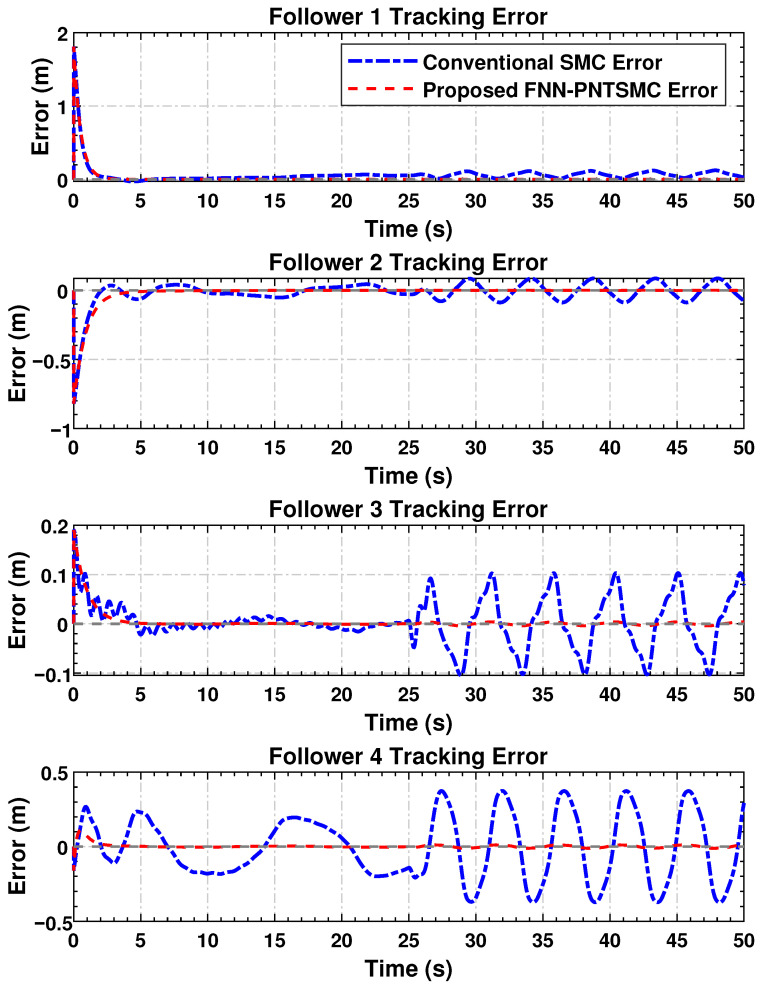
Synchronization errors’ evolution between follower agents indicating successful convergence in the multi-agent network.

The evolution of synchronization error for the follower agents is depicted in Figure 4. The standing drift in the magnitude of the errors provides additional evidence of the control algorithm’s capability in coordinating agents’ behaviors. Regarding settling time for mismatch convergence and disturbance attenuation, the proposed approach significantly outperforms SMC.

Error plots demonstrate that all agents converge rapidly, and the disparity values tend toward zero. Based on the simulation results, smoother error profiles with substantially reduced chattering are obtained using the proposed controller compared to the SMC reference. This confirms the successful operation of the adaptive FNN component in mitigating inter-agent discrepancies, even under time-varying disturbances and nonlinear uncertainties.

#### 4.4.5. Velocity Tracking

**Figure 5:**
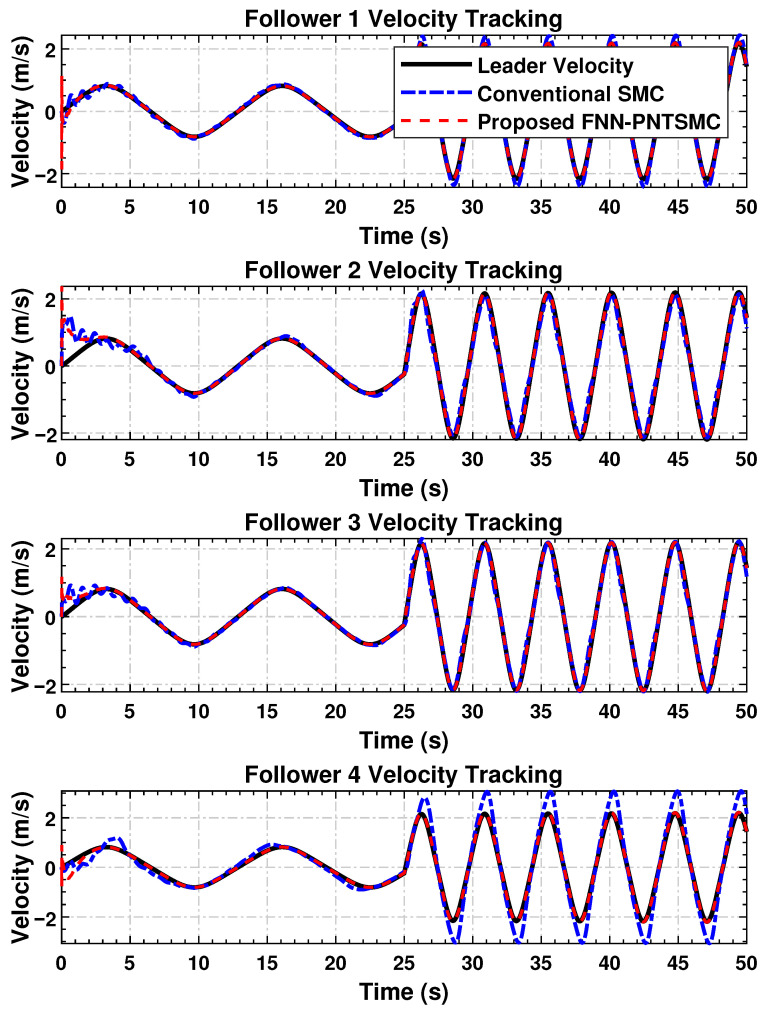
Velocity synchronization of the follower agents with the leader, demonstrating dynamic response under the control scheme.

The velocity profiles of the follower agents, reflecting the dynamic behavior of the system, are shown in Figure 5. The proposed control law ensures that each follower approaches the leader’s velocity smoothly and with minimal oscillation. In contrast to SMC, the proposed scheme yields superior transient performance and improved steady-state stability. The rapid corrective actions in the velocity response are well-damped, preventing abrupt fluctuations and excessive power loss—critical factors in systems operating under high inertia or rapid speed variations. The irregular and non-smooth transients observed under SMC control indicate sub-optimal regulation compared to the proposed method.

The transient overshoot in the followers’ velocity tracking arises from the strong corrective action of the prescribed-time control term $\frac{c_{i}}{{\left(T-t\right)}^{\delta }}$ and terminal sliding dynamics, which enforce rapid convergence within the fixed horizon *T*. This transient behavior reflects the trade-off between fast convergence and smoothness typical in prescribed-time and terminal SMC frameworks. The slightly higher overshoot under the proposed FNN-based controller results from the neural adaptation phase, where rapid online weight updates temporarily amplify control activity before accurate approximation of system nonlinearities is achieved. Once adaptation stabilizes, the controller ensures faster convergence, smaller steady-state errors, smoother control signals, and superior robustness compared to conventional SMC. Unlike fixed-gain SMC, the fuzzy neural adaptive NTSMC continuously tunes its parameters online, enabling improved long-term tracking under uncertainties. Moreover, the comparatively smoother performance of followers farther from the leader (e.g., follower 4) results from the directed communication topology, where intermediate agents naturally filter high-frequency transients during consensus propagation.

#### 4.4.6. Control Input Synchronization

**Figure 6:**
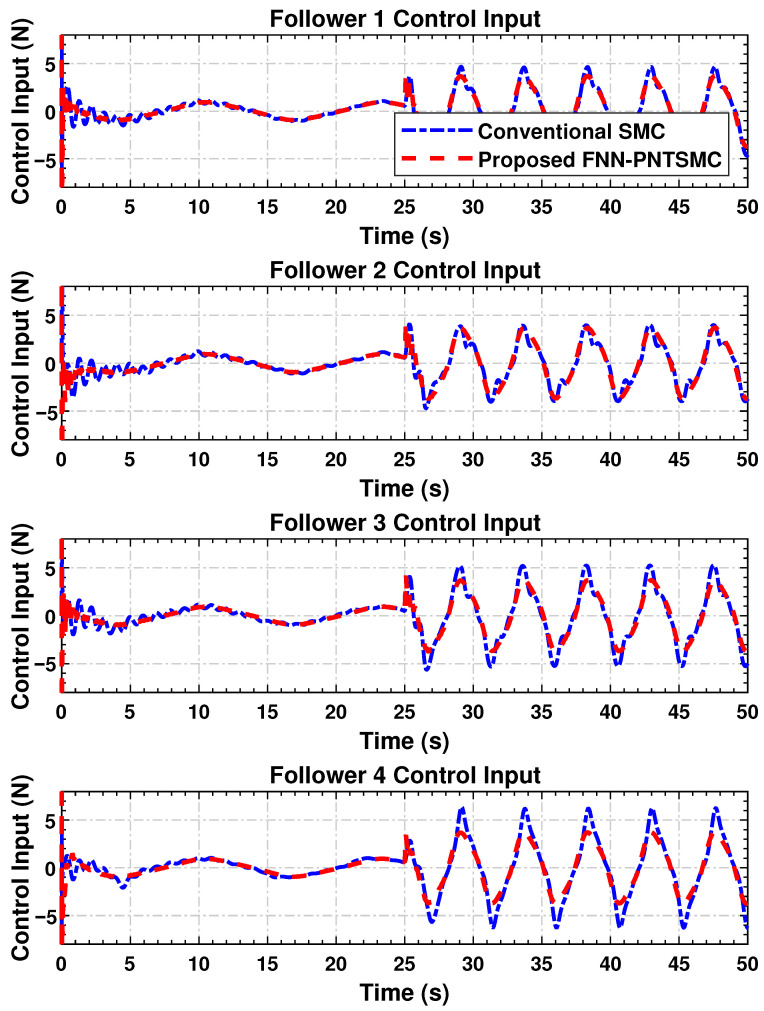
Time histories of control signals applied to follower agents, illustrating coordinated actuation and reduced chattering behavior.

The control signal trajectories are shown in Figure 6. These plots demonstrate that the control inputs of the agents are synchronizing each intra-agent signal with much less high-frequency switching. This is crucial in physical implementations, as it reduces the wear and energy burden in both electro-mechanical systems. Control profiles in that case are smooth and contain little sharp transition or spike. This indicates that the proposed controller is useful for smooth control, in contrast to the SMC scheme, which generates a discontinuous signal due to its discontinuity. There is no doubt that smoother input benefits systems that respond with physical actuators or those with bandwidth limitations, and this is one of our approach’s strengths.

The simulation results confirm the effectiveness of the proposed distributed control law in achieving high tracking accuracy (Figure 3), maintaining synchronization stability (Figure 4), regulating dynamic velocity and ensuring convergence (Figure 5), as well as delivering smooth and coordinated control efforts (Figure 6) under time-varying disturbances. Compared to the conventional SMC method, the proposed FNN-based adaptive controller demonstrates superior robustness, with significantly reduced steady-state and transient errors. Moreover, it achieves a substantial reduction in control forces. These findings suggest that the proposed controller performs reliably in practice and holds strong potential for application in multi-agent cooperative systems, robotic platforms, and precision networked control systems.

## 5. Conclusions

This work proposes a novel synchronization control strategy that integrates fuzzy neural networks with a prescribed-time nonsingular terminal sliding mode control framework for higher-order nonlinear multi-agent systems subject to uncertainties. The method guarantees that all follower agents align with the leader’s state within a predefined time bound, regardless of initial conditions, while effectively compensating for matched uncertainties under a directed communication topology. The FNNs provide real-time estimation of unknown nonlinear dynamics, and the terminal sliding surface is designed to eliminate singularities and ensure fast error convergence. A Lyapunov-based stability analysis verifies prescribed-time synchronization with bounded trajectories of the system. Simulation studies on a leader–follower network demonstrate superior robustness, accuracy, and adaptability compared to conventional sliding mode control techniques, confirming its potential for real-world deployment in uncertain and time-critical applications. Future research will focus on extending this approach to handle time-varying communication topologies, actuator faults, and heterogeneous agent dynamics, as well as conducting experimental validation to confirm its practical feasibility.

## Figures and Tables

**Table 1:** System, control design, and FNN parameters for leader and followers.

Leader Parameters		
Parameter	Symbol	Value
Mass	$m_{L}$	1.0 kg
Damping	$d_{L}$	0.5 Ns/m
Input Bound	$|u_{L}|$	≤10
Uncertainty Bound	$∥\Delta f_{L}∥$	≤0.2
**Follower Parameters**		
**Parameter**	**Symbol**	**Values for Followers 1–4**
Mass	$m_{i}$	[1.0, 1.2, 0.9, 1.1] kg
Damping	$d_{i}$	[0.5, 0.6, 0.4, 0.55] Ns/m
Input Bound	$|u_{i}|$	[≤ 10, ≤ 12, ≤ 9, ≤ 11]
Uncertainty Bound	$∥\Delta f_{i}∥$	[≤ 0.3, ≤ 0.35, ≤ 0.25, ≤ 0.28]
Prescribed Time	*T*	[5, 6, 4.5, 5.5] s
Sliding Gain	$k_{i}$	[3.0, 3.2, 2.8, 3.1]
Discontinuous Gain	$\eta_{i}$	[1.5, 1.6, 1.4, 1.55]
Terminal Power	$\alpha_{in}$	[0.7, 0.65, 0.75, 0.7]
Coupling Gain	$c_{i}$	[2.0, 2.2, 1.8, 2.1]
Singularity Parameter	δ	[1.2, 1.3, 1.1, 1.25]
Number of Rules	$N_{r}$	[7, 8, 6, 7]
Learning Rate	$\gamma_{wi}$	[10.0, 9.5, 10.5, 10.0]
Initial Weights	$W_{i}\left(0\right)$	Rand in specified ranges
Basis Function Type	–	Gaussian
Approx. Error Bound	$\epsilon_{i}$	[ ≤0.05 , ≤ 0.06, ≤ 0.04, ≤ 0.05]

## Data Availability

The original contributions presented in this study are included in the article. Further inquiries can be directed to the corresponding author.
